# Nutrition: Basis for Healthy Children and Mothers in Bangladesh

**DOI:** 10.3329/jhpn.v26i3.1899

**Published:** 2008-09

**Authors:** A.S.G. Faruque, A.M. Shamsir Ahmed, Tahmeed Ahmed, M. Munirul Islam, Md. Iqbal Hossain, S.K. Roy, Nurul Alam, Iqbal Kabir, David A. Sack

**Affiliations:** ICDDR, B, Mohakhali, Dhaka 1212, Bangladesh

**Keywords:** Child nutrition, Maternal nutrition, Nutrition disorders, Nutritional status, Protein-energy malnutrition, Bangladesh

## Abstract

Recent data from the World Health Organization showed that about 60% of all deaths, occurring among children aged less than five years (under-five children) in developing countries, could be attributed to malnutrition. It has been estimated that nearly 50.6 million under-five children are malnourished, and almost 90% of these children are from developing countries. Bangladesh is one of the countries with the highest rate of malnutrition. The recent baseline survey by the National Nutrition Programme (NNP) showed high rates of stunting, underweight, and wasting. However, data from the nutrition surveillance at the ICDDR, B hospital showed that the proportion of children with stunting, underweight, and wasting has actually reduced during 1984–2005. Inappropriate infant and young child-feeding practices (breastfeeding and complementary feeding) have been identified as a major cause of malnutrition. In Bangladesh, although the median duration of breastfeeding is about 30 months, the rate of exclusive breastfeeding until the first six months of life is low, and practice of appropriate complementary feeding is not satisfactory. Different surveys done by the Bangladesh Demographic and Health Survey, United Nations Children's Fund (UNICEF), and Bangladesh Breastfeeding Foundation (BBF) showed a rate of exclusive breastfeeding to be around 32-52%, which have actually remained same or declined over time. The NNP baseline survey using a strict definition of exclusive breastfeeding showed a rate of exclusive breastfeeding (12.8%) until six months of age. Another study from the Abhoynagar field site of ICDDR, B reported the prevalence of exclusive breastfeeding to be 15% only. Considerable efforts have been made to improve the rates of exclusive breastfeeding. Nationally, initiation of breastfeeding within one hour of birth, feeding colostrum, and exclusive breastfeeding have been promoted through the Baby-Friendly Hospital Initiative (BFHI) implemented and supported by BBF and UNICEF respectively. Since most (87-91%) deliveries take place in home, the BFHI has a limited impact on the breastfeeding practices. Results of a few studies done at ICDDR, B and elsewhere in developing countries showed that the breastfeeding peer-counselling method could substantially increase the rates of exclusive breastfeeding. Results of a study in urban Dhaka showed that the rate of exclusive breastfeeding was 70% among mothers who were counselled compared to only 6% who were not counselled. Results of another study in rural Bangladesh showed that peer-counselling given either individually or in a group improved the rate of exclusive breastfeeding from 89% to 81% compared to those mothers who received regular health messages only. This implies that scaling up peer-counselling methods and incorporation of breastfeeding counselling in the existing maternal and child heath programme is needed to achieve the Millennium Development Goal of improving child survival. The recent data showed that the prevalence of starting complementary food among infants aged 6-9 months had increased substantially with 76% in the current dataset. However, the adequacy, frequency, and energy density of the complementary food are in question. Remarkable advances have been made in the hospital management of severely-malnourished children. The protocolized management of severe protein-energy malnutrition at the Dhaka hospital of ICDDR, B has reduced the rate of hospital mortality by 50%. A recent study at ICDDR, B has also documented that home-based management of severe protein-energy malnutrition without follow-up was comparable with a hospital-based protocolized management. Although the community nutrition centres of the NNP have been providing food supplementation and performing growth monitoring of children with protein-energy malnutrition, the referral system and management of complicated severely-malnourished children are still not in place.

## INTRODUCTION

Protein-energy malnutrition is a syndrome resulting from interaction between poor diets and diseases, leading to anthropometric deficits and generally with deficits in micronutrients. Protein-energy malnutrition in children can be of three types by clinical classification: marasmus (wasting from malnutrition), kwashiorkor, and marasmic kwashiorkor; the latter two are oedematous malnutrition. Three anthropometric indices are commonly-used indicators of malnutrition: weight-for-age (underweight), height-for-age (stunting), and weight-for-height (wasting) (length is used if the age is less than two years or the length is less than 85 cm). A deficit (z-score below −2) in any one of these indices reflects malnutrition, and a z-score below −3 reflects a severe form of that condition.

In developing countries, an estimated 50.6 million children aged less than five years (under-five children) are malnourished, and those who are severely malnourished with a severe illness leading to hospitalization face a case-fatality rate exceeding 20% (1). In the early 1990s, using data from eight community-based, prospective studies conducted in Asia and Africa, Pelletier *et al.* estimated the relative risk for mortality associated with different degrees of childhood malnutrition as 2.5, 4.6, and 8.4 for mild, moderate, and severe malnutrition respectively (2). Recent data from the World Health Organization showed that 60% of all deaths, occurring among under-five children in developing countries, could be attributable to malnutrition, and the Global Burden of Disease Study estimated that childhood malnutrition alone accounted for approximately half (15.9%) of the global loss of disability-adjusted life years (DALYs) (3) (DALYs represent the sum of years of life lost from premature mortality and years lived with disability adjusted for severity). Thus, poor nutrition severely hinders personal, social and national development. Bangladesh has the highest prevalence of childhood underweight among all countries in the world, except North Korea, and only seven countries have a higher prevalence of child stunting (4).

Protein-energy malnutrition impairs the immune system, leaving malnourished children less able to combat common diseases, prolongs or exacerbates the course of an illness, heightens the adverse impacts of toxic substances, causes short stature and reduced physical work capacity, and increases the future risk of heart diseases. In addition to increasing the risk of death (2), severely-malnourished children are likely to have a lower intelligence level, behaviour problems, and poor school achievement. Impaired mental development is perhaps the most serious long-term handicap associated with early childhood malnutrition (5).

At the ICDDR, B hospital, severe malnutrition was observed as an associated underlying disorder contributing to the death of 74% of children admitted to the Special Care Unit of the hospital (6). Even after discharge from the hospital, the severely-malnourished children were found to have significantly higher death rates; the first three months after discharge being the most critical. About 70% of deaths occurred in that period and severely-malnourished children had a risk of death 14 times that of their well-nourished counterparts (7). Certain factors increase the risk of marasmus, including other siblings aged less than five years in the family, being female, and history of using milk substitutes (8,9). Higher maternal education reduced the risk. Bhuiya *et al*. found that severely-malnourished children in Bangladesh had a risk of death nine times that of their counterparts with better nutritional status (10).

In addition to being a direct health concern, nutrition is central to overall health, and, in fact, the nutritional status of children and women is likely the best indicator of overall well-being. In this paper, we discuss the nutritional trends in the country and highlight some key issues for deficient nutritional status. A separate paper [in this volume] deals with specific micronutrient deficiencies and anaemia.

## NUTRITIONAL TRENDS IN BANGLADESH

Globally, nutritional status is considered the best indicator of the well-being of young children and a parameter for monitoring progress towards the Millennium Development Goals (MDGs), especially MDG 1. This section updates and identifies the trends in childhood malnutrition using (a) nutrition surveillance information from a baseline survey of nutrition carried out for the National Nutrition Programme (NNP) in Bangladesh in 2004, (b) information from the surveillance system of the ICDDR, B hospital in Dhaka from 1979 (11), and (c) data from other surveys conducted recently.

Low birthweight (<2,500 g) is an especially important indicator: both as a marker of overall health of the mother and as a predictor of ill-health for newborns. Rates of low birthweight among Bangladeshi children are among the highest in the world with 30-40% of babies weighing less than 2,500 g at birth. It has been thought that improved nutrition for mothers during pregnancy would reduce this rate. Scientists at ICDDR, B are in the process of assembling the results of a very large nutrition intervention trial attempting to reduce rates of low birthweight. Results of the study are being analyzed.

**Key messages**

- Malnutrition is estimated to be an ‘underlying cause’ of about 60% of childhood deaths in Bangladesh. This attribution as an ‘underlying cause’ hides the observation that, if malnutrition had been corrected, the child would not have died.
- There have been slow improvements in overall nutrition indicators in Bangladesh.
- Exclusive breastfeeding is widely recommended, but poorly practised in Bangladesh. Many small-scale interventions have effectively improved rates of exclusive breastfeeding, but none has been effectively brought to scale.
- Children in Bangladesh ‘fall off the growth curve’ when they start to take complementary foods. Improving growth during this time will require efforts to improve complementary feeding behaviours. Families have been receptive to these changes when introduced through small-scale educational programmes.

**Fig. 1:**
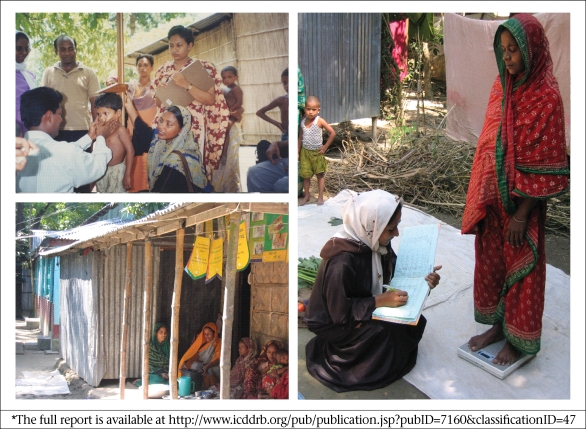
The baseline survey for the National Nutrition Programme*

The NNP commissioned ICDDR, B, in collaboration with the Institute of Public Health Nutrition and the National Institute of Population Research and Training (NIPORT) and with the assistance of Mitra and Associates, to carry out a baseline nutrition survey to assess various nutritional and socioeconomic indicators in a sample of intervention and control upazilas (subdistricts) in Bangladesh. [The intervention upazilas are those that were included in the Bangladesh Integrated Nutrition Project (BINP) which preceded the NNP.] The survey included data from 44 ‘new’ NNP upazilas, 53 ‘old’ BINP upazilas, and 16 control upazilas which were not scheduled for the intervention. This survey was conducted about two years after the BINP had functioning and just as the NNP was starting its activities. The photos show a typical community nutrition centre (CNC) where services are provided in a village through the NNP and the teams carrying out the anthropometry (Fig. 1). Although the survey was not designed primarily to compare different types of upazilas, there was a need to include a sample of upazilas from each category.

In total, 26,424 subjects from 151,372 household members participated in the survey. The survey collected data from under-five boys and girls, adolescent girls, and pregnant women. The primary variables included weight, height, and mid-upper arm circumference (MUAC) of under-five children; weight, height, and MUAC of pregnant women; weight gain in pregnancy; birthweight, anaemia among adolescent girls and pregnant women; and iodine status of adolescent girls and pregnant women.

Secondary variables included socioeconomic data of households, infants, and children: feeding (exclusive breastfeeding, complementary feeding), growth monitoring and promotion, and diet of adolescent girls. Data on pregnant women which included age, gravida, pregnancy-related complications, duration of pregnancy, antenatal check-up, rest during pregnancy, dietary practice, and plans for breastfeeding were collected. Information on micronutrients included the use of iodized salt, iodine content of table salt, micronutrient intake in food, intake of iron tablets by pregnant women, and intake of vitamin A capsules at delivery. Stool was collected to see the presence of ova of helminths.

Information was also collected regarding involvement in home-gardening, poultry, pisciculture, dairy farming, nursery and income-generating activities, issues of food security, and intake of protein-rich food and micronutrients, participation in adolescent girls’ forums, women's groups, non-formal education and training on skills development, nutrition knowledge, child care, dietary practices of adolescents and pregnant women, domestic hygiene, and healthcare practices.

**Fig. 2:**
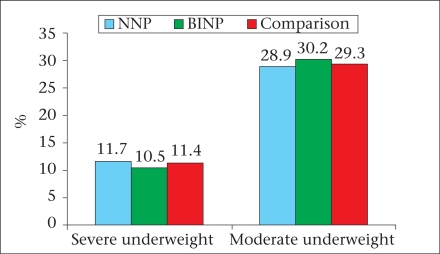
Underweight among under-two children

**Fig. 3:**
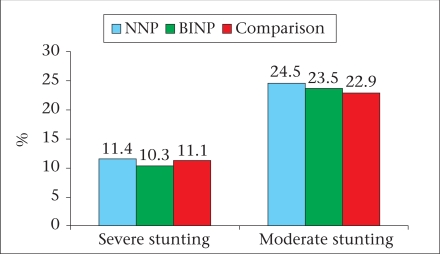
Stunting among under-two children

**Fig. 4:**
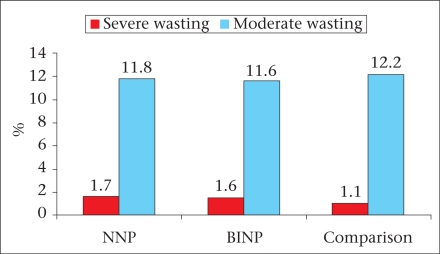
Wasting among under-two children

**Fig. 5:**
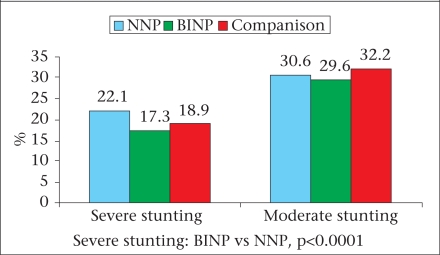
Stunting among 3-5 years old children

**Fig. 6:**
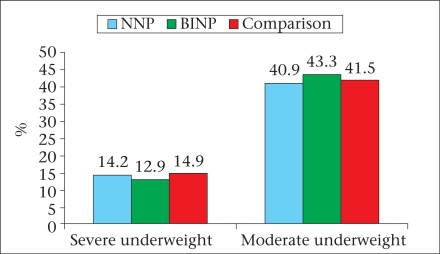
Underweight among 3-5 years old children

**Fig. 7:**
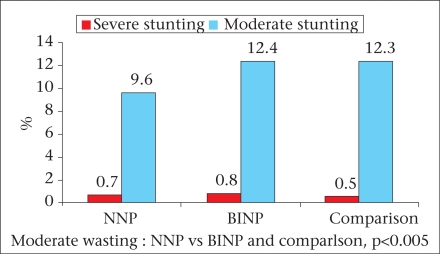
Wasting among 3-5 years old children

The major findings of the survey are briefly described here and are illustrated in figures 2-7. The age, weight, height, MUAC, height-for-age z-score, weight-for-age z-score, and weight-for-height z-score of 0-23 month(s) old children did not differ among different categories of upazilas. Also, the anthropometric indices of boys and girls in this age-group did not differ, even when age-groups were divided into smaller categories of 0-5 month(s), 6-11 months, and 12-23 months. There were some small differences in certain subcategories; however, these were relatively minor in importance from a clinical standpoint. The overall rates of mild, moderate and severe malnutrition are shown in the three graphs showing high rates of stunting, underweight, and wasting. As noted, moderate and severe stunting and underweight were both very common, although severe wasting was relatively rare, occurring in less than 2% of children in the <2 years age-group.

Among children aged 3-5 years, the results were similar, with high rates of moderate and severe stunting and underweight but low rates of wasting. Interestingly, for children aged 36-47 months, the mean height-for-age z-score was greater in the BINP areas compared to children in the NNP and control areas; this perhaps reflects the impact of the earlier BINP intervention.

**Key messages from the ICDDR, B hospital surveillance**

- Improving nutritional trends in this group which represent the poorest of the poor and the vulnerable.
- Concern about the plateau in severe wasting.
- Clear improvements in coverage of vitamin A and measles vaccine.

Consistent with previous surveys, the improved nutritional status of both infants and older children was benefited by increasing education of mothers and was worsened by measures of severe poverty. Girls were relatively more malnourished in the older age-group; however, the differences were less in those aged less than two years.

Although the children in the survey reflected a continuing and serious problem of malnutrition, the trends of underweight and stunting actually showed some long-term improvement. This is reflected in the graphs (Fig. 8A and 8B).

**Fig. 8A:**
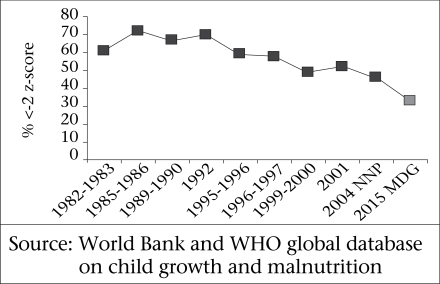
Prevalence of underweight among underfive children (<-2 z-score) in Bangladesh

**Fig. 8B:**
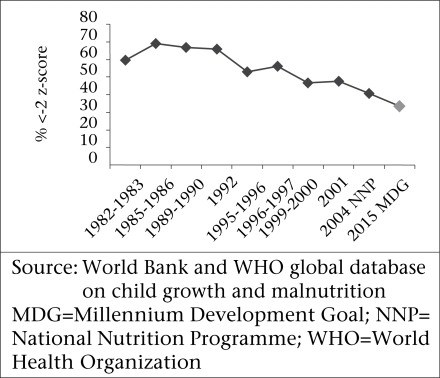
Prevalence of stunting (<-2 z-score) among under-five children in Bangladesh

With regard to the nutritional status, 35-38% of pregnant women had a body mass index (BMI) less than 18.5. Their average weight was 48-49 kg. Fewer than 40% had been receiving iron tablets during pregnancy, although over 50% had anaemia. The mean gain in weight during the last trimester was less than 4 kg, reflecting a continuing lack of calorie intake during pregnancy. The average birthweight was 2.8 kg, and about 20% of newborns had a birthweight less than 2.5 kg. (This rate of low birthweight is less than other recent surveys in Bangladesh.)

The average weight of adolescent girls was 41-42 kg (the mean age was 15 years). Iodine deficiency was still common. About 40% of household salts had low levels or no iodine, and about 40% of girls were deficient by urine test.

The survey showed that chronic malnutrition continues as a major problem in Bangladesh, and this involves deficiencies in both calories and micronutrients. However, there has been some progress, and several indicators from the baseline suggest that the BINP activities which are now incorporated into the NNP have resulted in some improvements, although these seem to be only slight improvements. Clearly, these indicators need to improve much more rapidly if Bangladesh is to achieve the MDGs relating to child health and nutrition.

## NUTRITION SURVEILLANCE AT THE ICDDR, B HOSPITAL

A surveillance system was established in 1979 at the Dhaka hospital of ICDDR, B which tracks the trends in patients coming for treatment at this hospital (12). This is a systematic sample of all patients (4% sample between 1979 and 1995, and a 2% sample since then), and clinical, epidemiological and microbiological information is recorded in an electronic database. From this database, we extracted data relevant to this analysis, including age, sex, pathogens identified, use of vitamin A capsules, immunization, and socioeconomic and nutritional indices. Patients coming to the hospital are not representative of the population of Bangladesh, but they do represent a ‘snapshot’ of the poorest of the poor, since this socioeconomic group makes up a large majority of patients coming to the ICDDR, B hospital, mostly from Dhaka.

**Fig. 9:**
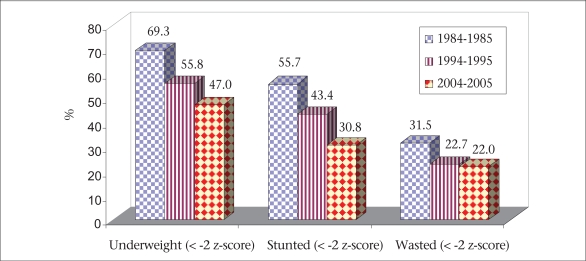
Changing trend in nutritional status

There has been a decrease in the proportion of children with stunting and severe stunting when analyzing data from three time periods: 1984–1985, 1994–1995, and 2004–2005 (Fig. 9). The rates of stunting decreased from 56% to 31% during this time period. Similarly, the rates of underweight decreased from 69% to 47%. For wasting, the decrease was from 31% to 22%. Severe wasting decreased from 8% to 4%, but has not trended downward further—a concerning development. We conclude that there has been a large reduction in moderate malnutrition over a 20-year period in these patient groups, although we remain concerned that rates of severe wasting remain high in this population of very poor persons living in Dhaka.

## BREASTFEEDING

The WHO estimated that promotion of exclusive breastfeeding for the first six months could avert the deaths of 1.3 million infants globally each year (13). This conclusion is based on earlier studies demonstrating that exclusive breastfeeding reduces morbidity and deaths from common infectious diseases (13-17). The risk of death from diarrhoeal diseases and pneumonia is, respectively, 14 and 4 times higher in bottlefed infants in developing countries compared to infants exclusively breastfed for the first 4-6 months of their lives (14). Breastfeeding exerts its strongest beneficial effect in children aged 1-15 month(s) (18). Since the 1980s, a number of global initiatives have been undertaken to increase the rate of exclusive breastfeeding, including the child-survival resolution by UNICEF, the World Declaration on Children, International Code of WHO for Marketing of Breast Milk Substitutes, the Baby-Friendly Hospital Initiative (BFHI), and the International Conferences on Nutrition. However, these global breastfeeding promotional activities are focused on improving practices in hospitals and other facilities (19). In Bangladesh, most mothers deliver at home, and these strategies are unlikely to have the desired impact.

### Magnitude and diversity of the problems

Although the prevalence of breastfeeding is very high in Bangladesh, appropriate breastfeeding is rarely practised. Infants are introduced to other foods either too early or too late. In some families, colostrum is discarded, and it is quite common to give prelacteal foods to the newborns and even to delay the onset of breastfeeding by more than 24 hours. Despite universal (97%) breastfeeding and a median duration of breastfeeding of over two years, the proportion with exclusive breastfeeding remains extremely low, and this is for a short duration (the median duration of only 1.8 months) (20). Studies from the 1990s reported exclusive breastfeeding up to four months by only 5-16% of both urban and rural mothers (21,22). The most recent Bangladesh Demographic and Health Survey (BDHS) observed a rapid decline in exclusive breastfeeding during the first six months of infants—from 63.5% in the first month to 30% during 4-5 months (23). This short duration of exclusive breastfeeding is related to early introduction of plain water and breastmilk substitutes, such as powdered milk and cow's milk, and semi-solid gruels.

The NNP of the MoHFW reported that 98% of 28,584 newborn babies were fed colostrum but only 37% of infants were exclusively breastfed for an average of five months in 58 subdistricts. This is similar to previous estimates, suggesting no improvement in the situation over the last decade.

**Fig. 10:**
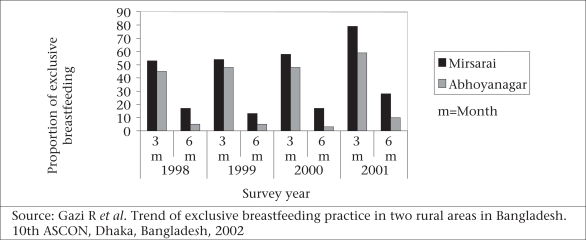
Proportion of exclusive breastfeeding in two field sites of ICDDR, B

**Fig. 11:**
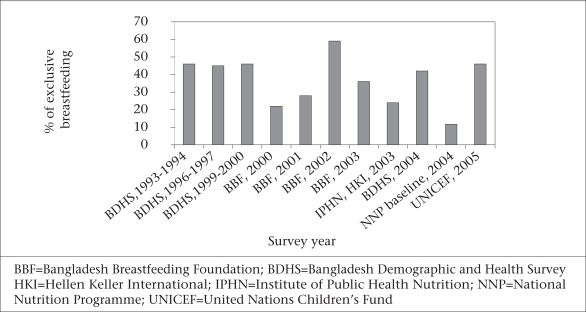
Proportion of exclusive breastfeeding infants from national surveys

Other data generated by ICDDR, B from two of its field areas (6,818 households in Mirsarai and 4,752 such households in Abhoynagar) demonstrated significant declines in the percentage of exclusive breastfeeding among infants with age during the first six months (Fig. 10). The differences in the proportions of exclusive breastfeeding presented in Figure 10 and 11 may result from the differences in the study design and definitions of exclusive breastfeeding, but these are consistent in showing no improvement in rates of exclusive breastfeeding.

In a study involving 1,100 lower-middle class mothers who received advice, 97% stated that they understood that exclusive breastfeeding included the provision of plain water (24). Although the messages about exclusive breastfeeding were well-received, the prevalence of exclusive breastfeeding in this study was only 15%, and complementary foods were introduced early, at a median of 30 days of life. Many mothers doubted the message that breastmilk alone is sufficient for a five-month old child.

### Interventions to improve breastfeeding practices

Considerable efforts have been invested in encouraging exclusive breastfeeding, including feeding colostrum to their babies, but with lesser emphasis on initiation of breastfeeding soon after delivery and discouraging prelacteals. Nationally, exclusive breastfeeding is promoted mainly through the BFHI and, to a more limited extent, through audiovisuals and mass media. Since most deliveries take place in home, the BFHI has no mechanism for continuing support to mothers after their discharge. The importance of continued support is illustrated by the observation of breast problems in 11% of women: such women are most likely to stop breastfeeding their children in the absence of any support (25).

### Counselling of exclusive breastfeeding

Routine health education appears to be ineffective in improving rates of exclusive breastfeeding, but appropriate counselling motivates mothers to implement and continue exclusive breastfeeding (20,26). One study evaluated the impact of counselling of mothers of young infants brought to the hospital for the treatment of diarrhoea by trained breastfeeding counsellors (20). On admission, all infants were either non-breastfed or partially breastfed, and at discharge, 60% of mothers counselled for exclusive breastfeeding versus only 6% receiving only routine health messages were motivated and were practising exclusive breastfeeding. Follow-up of these mothers two weeks later found that 75% of counselled mothers compared to 8% in the control group were practising exclusive breastfeeding. (The reason for this further increase in exclusive breastfeeding may be due to definitions used, since infants with mild diarrhoea receiving ORS were regarded as predominantly breastfed; when they went home after resolution of diarrhoea, they became exclusive breastfeeding mothers.)

One-on-one counselling, despite being effective, may not be cost-effective, so other strategies have been explored. One study compared individual vs group counselling and observed individual counselling to be more effective when initiated during pregnancy and continued for five months postpartum. More frequent and timely counselling resulted in higher rates of exclusive breastfeeding. A counselling schedule designed to have two contacts during pregnancy, two contacts soon after delivery when mothers are most likely to experience difficulties with breastfeeding, and as needed thereafter, was found to be very effective.

Another alternative, peer-counselling in the community provides an opportunity to interact with other family members involved in the decision-making processes (26). This is important in the Bangladesh context, where mothers generally live in extended families. Community-based studies on the effectiveness of peer-counselling in an urban community in Dhaka, Bangladesh, showed that trained community counsellors could significantly improve exclusive breastfeeding. In a study of middle-class urban community in Dhaka, pregnant women were identified during their last trimester of pregnancy and were counselled by peer-counsellors recruited from the same community. The peer-counsellors received the 40-hour WHO breastfeeding course and counselled mothers during pregnancy, within 48 hours of delivery, on the 5^th^ day, and between 10 and 15 days, and then monthly for five months. The rate of exclusive breastfeeding was 70% in the intervention group versus only 6% in the control group. Performance of peer-counsellors yielded similar results as trained counsellors.

There is a growing evidence that group counselling with less frequent contact between mothers and counsellors could be quite effective. A study compared the effectiveness of individual peer-counselling and group peer-counselling on exclusive breastfeeding in a rural community in Bangladesh (Kabir I. Personal communication, 2003). Three unions of a rural subdistrict in Chittagong district of Bangladesh, a conservative area with a low rate of female literacy, limited exposure to electronic and print media, and low contraceptive prevalence rate (CPR) were randomized. Local women from two unions were trained as peer-counsellors who provided support and counselling to pregnant women during the third trimester to practise exclusive breastfeeding. The study involved more than 10 counselling visits, and at the end of six months, the rates of exclusive breastfeeding were 89% and 81% among mothers receiving individual and group counselling respectively. The rate was only 12% among mothers in the control group who received routine health messages but no specific counselling. Both these randomized, controlled community trials have observed that early and repeated counselling contacts with mothers significantly increase the rate and duration of exclusive breastfeeding.

**Fig. 12:**
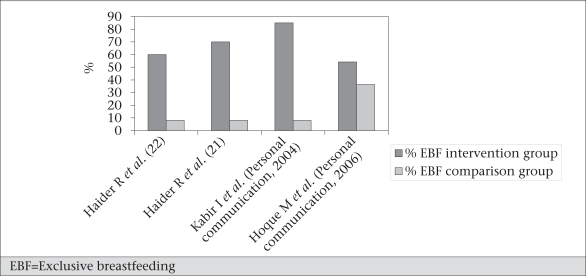
Effectiveness of peer-counselling on EBF in Bangladesh

Figure 12 shows the results of different studies on counselling for exclusive breastfeeding. The variations in results may be due to the variations in population intervened and in the intensity of the interventions. However, results of these studies indicate their potential to improve rates of exclusive breastfeeding (Table). A combination of one-on-one and group counselling was also found as effective as individual counselling in promoting exclusive breastfeeding, and this could be a more cost-effective approach. Although these different counselling strategies have proved to be effective, these have not yet been brought to scale.

**Table.** Effects of breastfeeding counselling on proportions of exclusive breastfeeding in developing countries

Author/reference	Sample size	Rate of EBF
Haider (27)	360	60
Morrow (28)*	170	67
Haider (22)	626	70
Bhandari (29)	1,075	49
Kabir (unpublished)	355	85

*Morrow's data are for 3 months, others' data for 6 months; EBF=Exclusive breastfeeding

### Integration of exclusive breastfeeding with other programmes

It seems unlikely that a stand-alone exclusive breastfeeding programme will be cost-effective; however, messages on exclusive breastfeeding can well be integrated into other programmes which are being scaled up in Bangladesh. These include Integrated Management of Childhood Illness (IMCI), Saving Newborn Lives Initiative (SNL), NNP, BFHI, and the Family Planning Programmes of the country. The exclusive breastfeeding messages should be reinforced with mass media and with reinforcement during EPI visits and other medical clinic contacts (prenatal visits and well-baby visits). A consistent approach through all these sources may well achieve results not possible with single programmes.

## COMPLEMENTARY FEEDING: THE BANGLADESH PERSPECTIVE

In developing countries, weight gain of children commonly falters between 3 and 15 months of age. From 15 months onward, no further deterioration has been observed (30). One of the primary explanations for poor growth of the child during this period is an insufficient or inappropriate dietary intake. To meet their physiological requirements, infants aged above six months require high-quality complementary foods in addition to breastmilk (31). In 1998, the WHO and UNICEF jointly published a document which defines complementary feeding as giving additional food when breastmilk alone is no longer sufficient to meet the nutritional requirements of the infant. Any non-breastmilk foods or nutritive liquids that are given to young children during this period are defined as complementary foods (32).

**Breastfeeding and complementary feeding**

- Exclusive breastfeeding for six months is rare in Bangladesh: this behaviour has not improved over the last two decades.
- Complementary foods start too soon and are unsafe.
- Complementary foods contain a little calories, proteins, or micronutrients.
- Families do respond to messages about improving diet of infants.
- Feeding behaviours and types of food are critical.

Important factors in the success of complementary feeding include the following:

- Age for introduction of complementary foods and optimal duration of breastfeeding*
- Sufficient energy and nutrients required from complementary foods
- Appropriate food consistency for age‡
- Safe storage and preparation$
- Care during feeding†

In general, recommended infant-feeding practices are not being followed in either the urban or rural communities in Bangladesh. After six months, insufficient food is given from the standpoint of energy, protein, and micronutrients. Furthermore, the most common feeding behaviour is not optimal for intake by the infant. Although a determination of trends in infant-feeding practices is not possible (there have been no systematic surveys of feeding behaviours), it is possible to understand some issues from a review of selected studies.

Complementary feeding generally starts too early or too late, and foods that are offered are often inappropriate (23). One longitudinal study of 110 infants investigated infant-feeding practices in a rural area from birth to one year of age (33). It was found that 100% of mothers breastfed their infants from birth to one year, almost every day, but bottles containing various kinds of milk and starchy food were added to 60% of diets of infants by three months and 80% by five months of age. This additional food was given mostly in diluted form, mostly tinned milk or powdered milk. Twenty percent of infants received full-strength cow's milk, but no powdered milk was given at full strength.

**Fig. 13:**
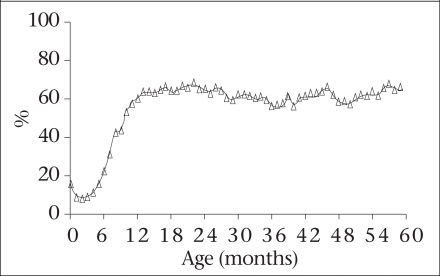
Prevalence of underweight among children aged less than 5 years in rural Bangladesh (1)

**Fig. 14:**
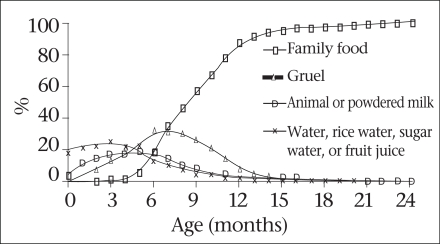
Common foods given to young children and infants aged <24 months in rural Bangladesh (n=26,567)

Results from a nutrition survey conducted by Helen Keller International showed that growth faltering arises when complementary foods are introduced in the diet of children (Fig. 13) (34). The findings of the survey suggest that the transition from breastmilk to family-food is very slow and that infants are usually given rice but are rarely given foods containing micronutrients and protein, even when these foods are available in the household. (Fig. 14) This suggests that there is the potential to improve diets of infants by encouraging households to give family-foods to infants.

There have been studies examining if families would change their feeding patterns in response to appropriate health education (36). Following recommendations on increasing the amount of food provided to the infant, the mean intakes from single meals increased from 40 g on day 1 to 64 g on day 7 (p<0.05). In the second trial, the mean meal frequency increased from 2.2 on day 1 to 4.1 on day 7 (p<0.05). The provision of high energy-dense diet, prepared by decreasing viscosity with α-amylase or by hand-mashing rice and *dal* into a paste before feeding, increased single-meal energy consumption from a mean of 54 kcal to 79 kcal or 75 kcal (<0.05) respectively. Both types of supplements were well-accepted and were used according to recommendations. It was concluded that it is possible to change short-term child-feeding behaviours to promote increased food intake.

## RECOMMENDATIONS TO IMPROVE COMPLEMENTARY FEEDING

Clearly, improvement in infant-feeding should be a major issue for improving the health of children in general in Bangladesh. It seems that families feed their children too little, too infrequently, and food that is primarily cereal-based with little protein and few micronutrients. Furthermore, the feeding pattern is not conducive to feeding, being most commonly ‘force-feeding’ as opposed to responsive feeding. Although poverty lies at the root of much of malnutrition in Bangladesh, for most families, nutritional intake could be improved with better feeding behaviours in the family.

An encouraging feature of small-scale studies is the responsiveness of families to respond to health-education messages. Feeding behaviours appear to be susceptible to change with proper involvement of the community. However, the messages regarding feeding behaviour have not been clear. Even the message for exclusive breastfeeding for six months is not widely practised, and the messages regarding complementary feeding and responsive feeding are not at all understood. Even when family-food is being consumed that includes a more balanced diet from animal sources and with micronutrients, small children tend to continue to receive only rice or other starches.

There is a clear need for improved nutrition messages through various media and to monitor these messages closely to ensure that these are clearly and fully understood. In addition, supplementation of micronutrients, e.g. zinc, iron, and vitamins, may need separate strategies. Although the scientific basis for appropriate complementary feeding is well-established, putting these data into practical programmes has been lacking.

## MANAGEMENT OF CHILDREN WITH SEVERE MALNUTRITION

When severely-malnourished children are hospitalized with an acute illness, the case-fatality rate of children receiving routine treatment in South Asia has often exceeded 20%. Ahmed *et al*. examined the benefits of a standardized protocol for the management of severely-malnourished children with diarrhoea admitted to the ICDDR, B hospital (1). Previously, this kind of child had a case-fatality rate of 17%; however, with the protocolized management, the case-fatality rate dropped to 9%, and only 40% of children in the test-group received intravenous infusions compared to 70% of the controls. This case-fatality rate has subsequently dropped even further to less than 5% at the ICDDR, B hospital (Ahmed T. Personal communication, 2007).

Because of the common fatal consequences of infection in children with severe protein-energy malnutrition, the WHO has produced a manual outlining this new protocolized management. The manual has subsequently been validated through training courses at ICDDR, B (37). Three phases of treatment are presented. First, children are rehydrated with oral or intravenous therapy, acute infections are treated with appropriate antibiotics, and trace elements are provided (but not iron). The daily dietary requirements should not exceed 80-100 kcal/kg of energy and 1 g of protein/kg. The second (rehabilitation) phase is initiated when appetite has been restored, and high-energy feeds with reasonable amounts of protein can be safely given with added iron. Once the acute complications of severe malnutrition are adequately controlled and children begin to eat avidly and start gaining weight, subsequent nutritional rehabilitation may be accomplished in a residential nutrition rehabilitation unit (NRU), in day-care centres, or at home.

Each of these approaches (NRU, day-care, and home-care) has certain advantages and limitations; so, local conditions need to be assessed to determine the most cost-effective approach. Studies in Bangladesh observed that, although the recovery rate (time needed to attain weight-for-height of 80% of the National Center for Health Statistics (NCHS) median) of very severely-malnourished children treated for one week in an inpatient ward followed by home management was somewhat longer (median=35 days) than the recovery rate of those treated in a specialized residential unit (median=18 days), the cost of home-treatment was less than one-fourth that of hospital-based care, and mothers preferred the home management (38,39). The same investigators also reported that, during a one-year follow-up period, children treated at home had less morbidity (episodes of cough and fever) compared to children managed in the residential unit, although other morbidities were similar (40). Heikens *et al*. compared early discharge vs continued hospital-based rehabilitation of malnourished Jamaican children and found that hospital treatment (mean stay of 40 days) resulted in greater weight and length at discharge and in the next few months compared to short-stay patients (mean stay of 18 days) (41). However, during the ensuing months and years, both groups moved towards the same levels of nutritional status expected in their home community.

A recent study at ICDDR, B (Ahmed T. Personal communication, 2007) compared the efficacy of home-based management with home-visits by study staff vs home-based management with outpatient follow-up vs full management at the NRU. Of 225 severely-malnourished children, the rate of weight gain was the lowest in the outpatient follow-up group, but there were no significant differences between the home-treated group with home-visits by staff and the NRU group in the rate of weight gain or in the number of days required to achieve a weight-for-height of >80% of the reference.

Although each of the above-mentioned studies found beneficial effects with community-based management of malnourished children with frequent follow-up, the high cost of these frequent home-visits may preclude large-scale implementation of this approach. It may be that similar benefits could be achieved if the follow-up assessment and supervision of treatment could be held at community clinics.

Since the facility-based management centre for severe child malnutrition is limited and expensive, Osiniski *et al*. developed and tested an approach for the identification and management of severely-malnourished children through routine primary-level outpatient clinics (42). During a 25-month enrollment period (2001–2003), 465 severely-underweight children (aged 6-23 months) were identified when attending NGO-run primary healthcare clinics serving the urban poor. Protocolized management included counselling on a home-prepared energy-dense and nutritionally-adequate diet, an eight-week course of micronutrients, outpatient treatment of any infectious diseases, scheduled monitoring of improvements of nutrition status, assisted referral for facility-based management if referral criteria were met and, for children from households with acute food insecurity or compromised caring capacity, provision of an energy-dense take-home supplement. Sixty-three percent (n=292) of enrolled children improved to moderate underweight, with a mean weight-for-age improvement between enrollment and discharge of 0.86 z-score. After a six-month follow-up, 44.7% of recovered children could be found, and 84.2% of them had sustained and even further improved on their weight-for-age at discharge. These findings suggest that protocolized management is effective for rehabilitating the majority of enrolled children from severe to only moderate underweight and that gains in nutrition status are sustained. Active outreach should, however, be incorporated in the protocol so as to increase community-level impact and to incorporate a preventative approach.

Since there are considerable constraints in terms of the number of hospital beds available to treat acutely-ill, severely-malnourished children, ICDDR, B recently evaluated a day-care strategy (43). Severely-malnourished children, aged 6-23 months, who had been refused admission to hospital due to lack of available beds, were managed at the Radda MCH-FP Centre in Dhaka, Bangladesh. Each day, parents brought their children to the clinic at 8:00 am and took them back home at 5:00 pm where they were treated according to the protocolized management for severe malnutrition. During February 2001–May 2003, 264 children were enrolled. There were no incidence of death during the acute phase of the day-care stay; however, one child died of severe pneumonia on day 12, and five other children died during the six-month follow-up phase. Results of this study indicate that severely-malnourished children can be successfully managed at existing day-care clinics, provided adequately-trained and motivated staff and support facilities are available at the clinic, thus providing an alternative to more expensive hospitalized care.

A modification in the management of severe malnutrition is through the use of ready-to-use therapeutic (RUTF). Using this approach, a series of 20,976 cases of severe acute malnutrition were treated in 21 community-based therapeutic care (CTC) programmes operating in Malawi, Ethiopia, and Sudan. Recovery rate of 78.1% and mortality rate of 4.3% were achieved with this approach (44). In Bangladesh, Concern Bangladesh tested the same CTC model in Khulna City Corporation through two local NGOs using a locally-produced food product (chickpea/sesame seed/dried skimmed milk/sugar/oil/cocoa-powder fortified with minerals and vitamins) (45). The initial findings showed that RUTF appeared to be highly acceptable. Although RUTF is mainly used in disaster situations, results of this pilot study in Bangladesh indicate that the CTC-RUTF strategy may be useful in Bangladesh under certain circumstances.

Results of other recent studies from Bangladesh showed that health and nutrition education (46), food supplementation with or without amylase-rich flour (47), and psychosocial stimulations (48) have very positive effects in the management of children with proten-energy malnutrition.

### Next steps needed in the management of children with severe protein-energy malnutrition

The WHO guidelines with or without minor modification according to the local context have played a valuable role in the management of children with severe protein-energy malnutrition. However, the current practice and coverage of these guidelines across the world, especially in Bangladesh, is very poor. One of the urgent goals of improved child survival should be incorporation of the guidelines into medical and nursing curricula and into in-service training programmes.

Although the Community Nutrition Centres of the NNP in Bangladesh have been providing food supplementation and performing growth monitoring of children with proten-energy malnutrition, the referral system and management of complicated malnourished and severely-malnourished children are still not in place. It is urgent to establish a proper referral system between Community Nutrition Centres and the nearest healthcare centre run by the Government or NGOs and to develop a standardized cost-effective health system to help these children who have a severe but relatively easily-treatable illness.

## Footnotes

*Should have exclusive breastfeeding from birth to six months of age and then introduce complementary foods (at 180 days) while continuing breastfeeding on demand until two years of age or beyond (35)

‡Ability of children to chew and swallow different forms of foods varies with age. In general, infants aged 6-11 months can consume soft and mashed foods; on the other hand, older children can consume some solid foods as done by other family members. Approximately at eight months of age, finger food (foods that a child can eat alone) should be introduced (32)

$Practice of good hygiene and proper food-handling by (a) washing hands of caregivers and children before food preparation and eating, (b) storing foods safely and serving foods immediately after preparation, (c) using clean utensils to prepare and serve food, (d) using clean cups and bowls when feeding children, and (e) avoiding the use of feeding-bottles which are difficult to keep clean (33)

†It is well-recognized that, besides the dietary aspects of feeding, behavioural interactions of the child-caregiver are also important for optimal nutrient and energy intake
